# Clinical and economic impact of an alert system in primary care for the detection of patients with chronic hepatitis C

**DOI:** 10.1371/journal.pone.0260608

**Published:** 2021-12-20

**Authors:** Antonio García-Herola, Raquel Domínguez-Hernández, Miguel Ángel Casado

**Affiliations:** 1 Digestive Medicine Section, Hospital Marina Baixa de la Vila Joiosa (Alicante), Marina Baixa de la Vila Hospital, Joiosa, Alicante, Spain; 2 Pharmacoeconomics & Outcomes Research Iberia (PORIB), Madrid, Spain; Centers for Disease Control and Prevention, UNITED STATES

## Abstract

**Introduction:**

Prevalence of chronic hepatitis C (CHC) is higher in patients born between 1955–1975. The aim was to perform an economic evaluation of an age-based electronic health record (EHR) alert in primary care to detect patients with undiagnosed CHC and its treatment in comparison with non-use of the alert system, in Valencian Community, Spain.

**Materials and methods:**

Decision trees and Markov model were used to evaluate the diagnosis and progression of the disease, respectively. CHC was diagnosed by serology and viral load in seropositive subjects. Epidemiological data and diagnostic costs were extracted from public sources of the Valencian Community. Probabilities, utilities and costs of model states were obtained from the literature. The impact on mortality and hepatic complications avoided by the implementation of the alert were estimated, and efficiency was measured as an incremental cost-utility ratio (ICUR) based on quality-adjusted life years (QALYs) and the costs of both alternatives.

**Results:**

The EHR alert detected 269,548 patients, of whom 1,331 had CHC (vs. 23 patients with non-alert). Over the patients’ lifetime, the alert would prevent 93% of decompensated cirrhosis cases, 87% of hepatocellular carcinomas, 90% of liver transplants, and 89% of liver related deaths compared to non-use of the alert system. In addition, it would obtain an additional 3.3 QALY per patient, with an incremental cost of €10,880 and an ICUR of €3,321.

**Conclusions:**

The implementation of an age-based EHR alert in primary care to detect patients with CHC reduces hepatic complications and mortality and is an efficient strategy.

## Introduction

Hepatitis C is a public health problem that affects millions of individuals around the world and causes the death of a large number of people [1,2]. Hepatitis C virus (HCV) is the most common chronic viral liver disease in Europe. It is transmitted primarily through blood and is related to the use of injected drugs and uncontrolled transfusions; to a lesser extent, it is also sexually transmitted. It is associated with different risk factors, including infection with other viruses, such as hepatitis B virus and human immunodeficiency virus (HIV); intravenous and nasal drug use; men who have sex with other men; blood transfusion history prior to 1992; and surgery prior to 1980 [3].

The epidemiology of the infection varies according to age. In Spain, the results of the most recent seroprevalence study show that in the general population from 20 to 80 years, there is a weighted prevalence of HCV antibodies of 0.85% (CI 95%: 0.64% -1.08%) and a weighted prevalence of active infection of 0.22% (95% CI: 0.12% -0.32%), with the highest prevalence among those born between 1948 and 1967 [4]. In the Valencian Community, these rates are higher, with an HCV seroprevalence of 1.1% and a prevalence of active infection at 0.4% [5].

Currently, a vaccine for hepatitis C is not available, but there are antiviral regimens based on direct-acting antivirals (DAAs) with high effectiveness shown in real life and which are also short and very well tolerated. In this sense, DAAs achieve response rates greater than 95% [6], suggesting that they eliminate the virus in practically all patients receiving treatment. Due to the progress achieved by these drugs, the World Health Organization (WHO) has proposed as a priority objective the elimination of hepatitis C as a health problem by the year 2030 [1]. To achieve this objective, it is essential to implement initiatives aimed at the active search for asymptomatic patients who do not know their state of infection and may develop complications derived from disease progresses.

The consequences of undetected infection can be evaluated in two different ways: from the clinical point of view and from the economic perspective. From the clinical point of view, a person who has the infection can remain asymptomatic for years, eventually progressing to liver cirrhosis (15–30%) [1], developing hepatocellular carcinoma, may need a liver transplant and even dying as the consequence of the development of hepatic and extrahepatic complications [1]. Additionally, viraemic patients can transmit the infection, especially if they do not know that they are infected, placing other people at risk. In terms of the economic perspective, the economic burden of HCV infection is high. The healthcare costs of chronic patients caused by the development of hepatic complications mentioned above are paid for by the National Health System (NHS) for years [7–9]. In addition, the disease generates very significant labour productivity losses, with the consequent economic impact [10].

In Spain, although different measures have been taken to detect hidden infection as of the implementation of the Global Strategic Plan for the Management of Hepatitis C in the Spanish National Health System (Plan Estratégico para el Abordaje de la Hepatitis C en España, PEAHC) in 2015, there is still a high percentage of undiagnosed population. These subjects belong to older age groups, usually greater than 40 yr. old [5]. Therefore, the development of actions to identify the infection in this population is a public health priority.

The objective of this study was to evaluate the clinical and economic impact of an age-based electronic health record (EHR) alert in primary care aimed at the age group with the highest prevalence of CHC, that is, those born between 1955 and 1975, to detect patients with CHC previously undiagnosed followed by DAAs treatment compared to non-application of the alert.

## Materials and methods

A cost-utility analysis was performed to evaluate the effectiveness, measured in terms of health outcomes, and the implementation costs of an EHR alert in primary care to detect undiagnosed patients with CHC and the subsequent administration of DAAs. The comparison condition consisted of the non-use of the alert, that is, the performance of occasional screenings according to routine clinical practice to detect hepatitis C in primary care.

The perspective adopted in the analysis was that of the NHS, the funding source for the implementation of the alert strategy and for the treatment of the patient. The study population comprised all residents of the Valencian Community with a health card who were born between 1955 and 1975, attended primary care and had never been previously diagnosed with hepatitis C (Table 1).

**Table 1:** Population parameters, clinic characteristic and unitary cost.

**Parameters**	**Valor**
Population Valencian Community (>18 years)	4,049,855 [11]
Population (1955–1976)	1,581,117 [11]
Population 1955–1976 with health card	1,540,493*
Patients who visit primary care	75%*
Not diagnosed	23% [5]
Serology (Alert vs No alert)	100%* vs 1.7%^†^
Anti-HCV (+)	1.14% [5]
Viral Load (+)	43.3% [5]
**Fibrosis stages**	
F0	23.8%*
F1	33.5%*
F2	16.8%*
F3	9.7%*
F4	16.1%*
**Health resources**	
HCV serology	€3.6 [18]
PCR	€35.8 [18]
FibroScan	€39.0 [18]
Abdominal ultrasound	€54.6 [18]
Blood analysis	€23.3 [18]
Primary care visit	€30.2 [17]
Hospital care visit (the first)	€67.4 [17]
Hospital care visit (subsequent)	€41.5 [17]

*Official data of the Autonomous Community.

^†^Assumption.

HCV, hepatitis C virus; PCR, Polymerase Chain Reaction.

**Fig 1:**
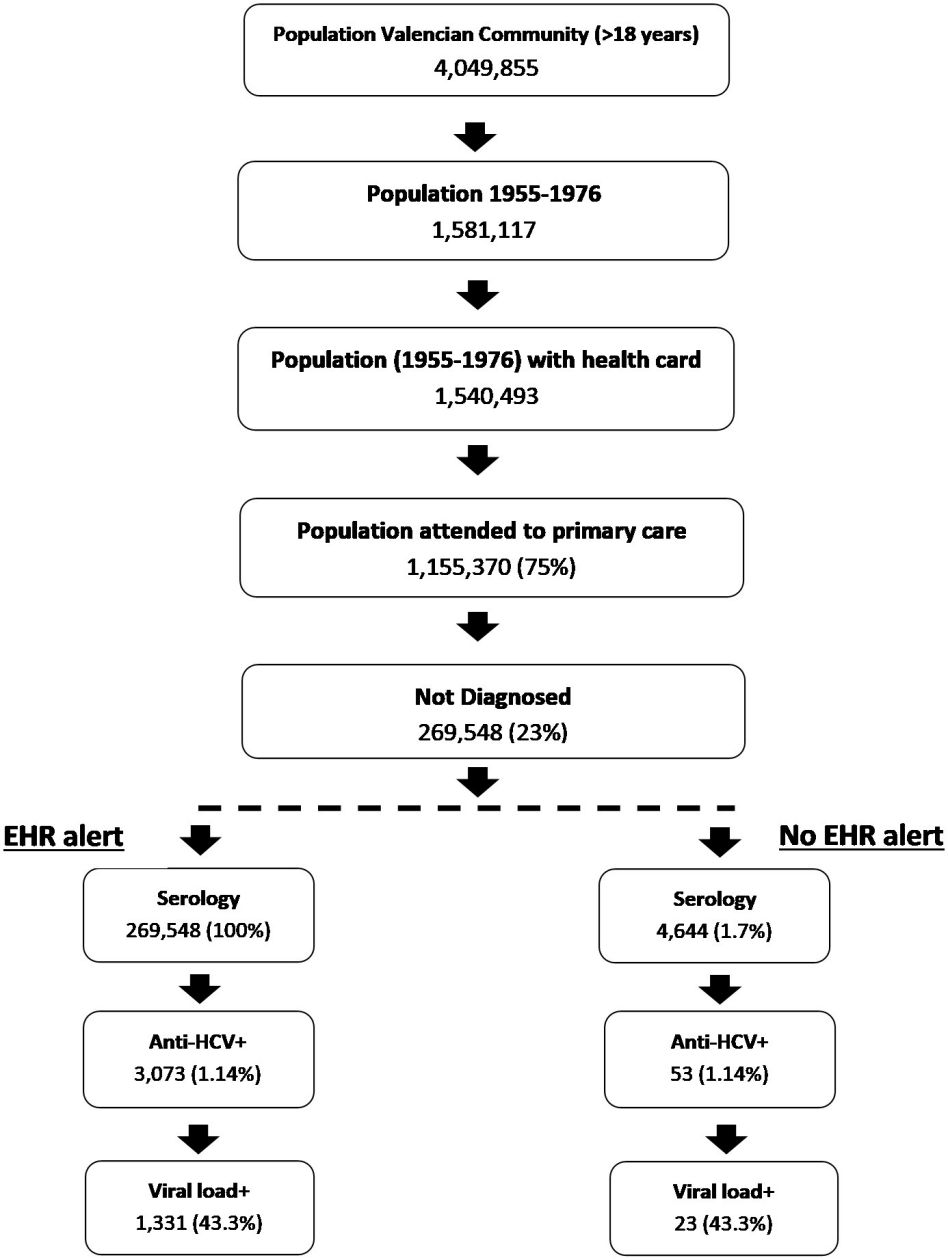
Population result flowchart. HCV: Hepatitis C virus; EHR: Electronic health record.

**Fig 2:**
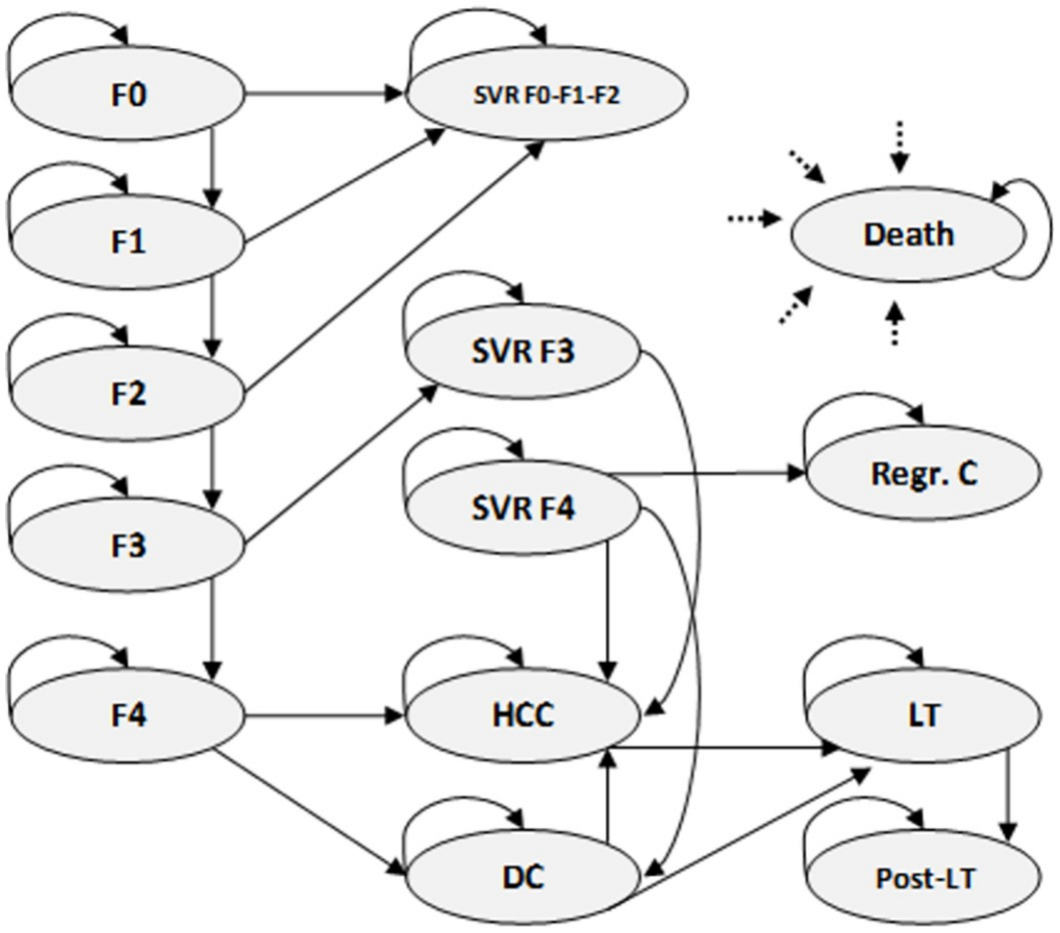
Markov model. State transition diagram. Patients enter the model based on their fibrosis state and can move through the different mutually exclusive health states at the end of each annual cycle or remain in the same health state, except for LT, where patients only remain for one cycle. Patients in SVR stages F0, F1 and F2 are considered cured patients and continue in that state until their death. Patients in SVR stages F3 and F4 remain at risk of developing HCC, and, in the case of SVR F4, are also at risk of DC. DC: Decompensated Cirrhosis; HCC: F: Fibrosis stage; Hepatocellular carcinoma; LT: Liver transplant; Post-LT: Post-Liver transplant; Regr. C: Regression of hepatic cirrhosis; SVR: Sustained Virologic Response.

The analysis was performed using an analytical decision-making model and was carried out in two phases: a) development of a decision tree to estimate the number of chronic patients detected with and without the implementation of the alert was estimated, based on the prevalence of the infection, and with the structure reflected in the flowchart presented in Fig 1; and b) the application of a Markov model adapted from previous studies to simulate the progression of the disease in patients with CHC (Fig 2) [8,9].

The demographic and epidemiological data of the population included in the decision tree, as well as the distribution of the patients according to their stage of hepatic fibrosis, were obtained from sources from the Valencian Community [11,12] or through information provided by experts (Table 1). The consumption of health resources considered for the diagnosis of the infection included the first visit a primary care, HCV serology and the determination of the viral load by PCR (Polymerase Chain Reaction) to HCV-positive subjects. On the other hand, the individuals identified who presented positive viremia, the visit to collect the results and referral from primary to specialized care, visits to specialist (hepatologist or infectious disease specialist) to complete the process of diagnosis, staging of the disease (analytical studies, liver elastography and liver ultrasound) and its treatment with DAAs, were considered.

The natural history of the disease was projected using a Markov model [8,9] composed of different health states defined by the different stages of liver fibrosis: mild fibrosis (F0, F1), moderate fibrosis. (F2, F3) and cirrhosis (F4) and the states of sustained viral response (SVR) for every stage, decompensated cirrhosis (DC), hepatocellular carcinoma (HCC), liver transplantation (LT) and death (Fig 2). Patients with CHC, once detected, were distributed within the model based on available information regarding the percentage of patients at each stage of fibrosis and cirrhosis. At that time, it was assumed that all patients diagnosed with CHC were treated with DAAs and that the SVR rates obtained were those associated with each state of fibrosis (95% SVR F0, 98% SVR F1, 97% SVR F2, 96% SVR F3, 94% SVR F4) [6]. The transition probabilities associated with each annual cycle of the simulation in the model were obtained from the published literature (Table 2) [8,9] and were established as dynamic probabilities by adjusting by age for each cycle of the model as a function of mortality by all causes.

**Table 2:** Parameters used in the Markov model.

**Health states**	**Transition probabilities** [8,9]	
F0 a F1	0.131	
F1 a F2	0.080	
F2 a F3	0.133	
F3 a F4	0.134	
F3 a HCC	0.011	
RVS F3 a HCC	0.003	
F4 a DC	0.040	
F4 a HCC	0.015	
SVR F4 a DC	0.003	
SVR F4 a HCC	0.006	
SVR F4 a Regr, C	0.055	
DC a HCC	0.068	
DC a LT	0.023	
HCC a LT	0.040	
LT a Post-LT	1.000*	
**Health states**	**Liver-related death** [8,9]	
DC	0.138	
HCC	0.430	
LT	0.210	
Post-LT	0.057	
**Health states**	**Utilities** [8,9]	**Costs (€)** [8,9]
F0	0.98	€272
F1	0.98	€272
F2	0.92	€315
F3	0.79	€315
F4	0.76	€573
SVR F0 y RVS F0	1.00	€116 (first year); €0 (second year and subsequent)*
SVR F2	0.93	€116 (first year; €0 (second year and subsequent)*
SVR F3	0.86	€116
SVR F4	0.83	€166
Regr, CH	0.86	€116*
DC	0.69	€2,332
HCC	0.67	€8,884
LT	0.50	€125,294
Post-LT	0.77	€36,623 (first year); €18,311 (second year and subsequent)

*Assumption.

DC: Decompensated cirrhosis; HCC: Hepatocellular carcinoma; SVR: Sustained virological response; Regr. C: Cirrhosis regression; LT: Liver transplant.

The time horizon considered in the analysis was the patient’s entire life once CHC was diagnosed. A discount rate of 3% to health costs and outcomes was applied [13].

The outcome measures were established in terms of effectiveness as the number of CHC patients detected and treated when comparing the implementation of the EHR alert to the non-use of the alert (obtained from the decision tree) and the difference in the life-years gained (LYG), quality-adjusted life years (QALY) and number of cases of hepatic complications avoided (obtained from the Markov model). Likewise, the total costs associated with the diagnosis, treatment and management of the disease were obtained for both strategies (alert and not alert). Efficiency was expressed as the incremental cost-utility ratio (ICUR) of the alert system compared to the non-implementation of the alert by dividing the total incremental costs by the QALY gained. The implementation of the EHR alert would be considered efficient if the result obtained was below the willingness-to pay (WTP) threshold commonly used in Spain, which ranges between €22,000 and €30,000 per QALY [14,15].

### Mortality

Patients with CHC may die from complications derived from advanced liver disease or from other causes. Therefore, in the analysis, the mortality associated with each health state was taken into account. The states of mild and moderate fibrosis were related to mortality by all causes by age obtained from statistical data for the Valencian Community [16], and data on mortality from advanced stages of the liver disease was obtained from the literature (Table 2) [8,9]. In addition, for these states, mortality from other causes was calculated excluding liver mortality.

### Utility values

Utility is a factor that adjusts LG and corrects them to QALYs. Utility values between 0 and 1 (with 0 indicating the worst health and 1 the best health) reflect the quality of life perceived by patients in the different phases of the disease. In the analysis, the annual utility values applied to each health state were obtained from the literature (Table 2) [8,9].

### Costs

As the incorporation of the alert system depends on the NHS, the costs incorporated into the analysis were direct health costs: those associated with the detection and diagnosis of the disease (medical visits, HCV serology and viremia and tests for liver disease staging) (Table 1) [17,18], treatment (pharmacological administration and monitoring of the effects of treatment on every patient) [8,9,19] and disease management associated with the different health states included in the model [8,9]. The cost of the design, programming and implementing the EHR alert system was considered zero as it is taken on by the NHS. The average pharmacological cost of the antiviral treatment per patient (€17,126) was calculated according to the total number of patients treated and the total investment in antivirals for HCV [6,19]. The costs were expressed in 2018 euros. Thus, the costs obtained from the literature were updated to 2018 values as a function of variations in the Consumer Price Index [20] (Table 2).

### Alternative scenarios

An “alternative analysis 1” was performed without including the cost of the first visit to primary care, considering that HCV screening is made in those patients who come to consultations for any other reason and not to potential hepatitis C infection.

In the “alternative scenario 2”, the potential uncertainty related to the detection of patients with undiagnosed CHC is evaluated, considering different percentages of screened patients (25%, 50% and 75%) using the EHR alert system, because the performance of serology depends on the involvement and participation of physicians and the acceptance of patients.

### Sensitivity analysis

To evaluate the uncertainty associated with some parameters, univariate deterministic sensitivity analyses (SA) were performed in which the following parameters were modified: prevalence of HCV antibodies (from 0.73 to 1.55%) [21], positive viral load (from 31 to 66%) [22], percentage of patients treated (from 100 to 90%), cost of the visit to the primary care doctor (from €18 to €42), and pharmacological cost of DAAs (± 60%). On the other hand, multivariate deterministic SA were performed modifying the percentage of F4 patients (from 16 to 33%) and thus varied the rest of the fibrosis states proportionally, the associated with the management of the disease costs (±20%) and the utilities [8,9].

## Results

Based on the results obtained with the decision tree, with the implementation of the age-based EHR alert for the population group born between 1955 and 1975, 269,548 patients without previous HCV serology would be tested, whilst only 4,644 patients would be screened without the alert, which implies a greater detection of CHC patients with the use of the EHR alert (1.331 vs. 23) (Fig 1).

Health outcomes in terms of the number of avoided cases of liver complications show a very significant reduction of greater than 83% in all cases when the implementation of the EHR alert system was compared to the non-application of the alert (Table 3).

**Table 3:** Health and economic results of the main scenario.

	EHR Alert	No EHR Alert	Difference (EHR alert vs No EHR alert)
**Economic results**			
Diagnosis (per chronic patient)			
*Analytics*, *tests*, primary and hospital care visit*	€7,224	€124	**€7,099**
Treatment			
*Pharmacological*	€17,126	€295	**€16,831**
*Treatment monitoring*	€262	€5	**€258**
Disease management			
*Liver complications*	€1,844	€15,152	**-€13,308**
**Total cost**			**€10,880**
**Health outcomes**			
LYG	18.8	16.8	**2.0**
QALYs	17.8	14.5	**3.3**
**Incremental cost-utility ratio (ICUR = Total cost/QALYs)**			**€3,321**
**Health results**			
Number of cases of liver complications of total chronic patients (1.331 patients)			
*Decompensated cirrhosis*	25	378	**-352 (-93%)**
*Hepatocellular carcinoma*	40	298	**-258 (-87%)**
*Liver transplant*	5	55	**-50 (-90%)**
*Liver-related deaths*	51	476	**-425 (-89%)**

*Includes first visit to primary care and serology, in addition to seropositive subjects, PCR, second visit to PC to confirm the result and referral to the specialist, specialist visits until the time of prescription of treatment, complete analysis, liver elastography and ultrasound abdominal. Design, programming and implementing (technology costs) of EHR alert was zero.

EHR: Electronic health record; LYG: Life-year gained; QALYs: Quality-adjusted life year.

Considering the total number of patients with CHC diagnosed (1,331 patients), the total cost of diagnosis for each patient with CHC, including all the analytics, tests, and visits of primary and specialist care until the time of the prescription of treatment, was €7,224 with the EHR alert system (detection of 100%) versus €124 non-application of alert (1.7% detection).

The results associated with the cost-utility analysis showed that detecting and treating the entire cohort of patients with CHC diagnosed by the EHR alert was associated with an increase of 2.0 LYG and 3.3 QALY per patient compared to non-application of the alert. Implementation of the alert had an incremental cost of €10,880; this represents an ICUR of €3,321 for each additional QALY gained (Table 3), which is below the generally accepted efficiency threshold. In the alternative scenario, not including the cost of the first visit to primary care would reduce the ICUR to €1,487 per QALY.

In the alternative scenario 1, without including the cost of the first visit to primary care, the total cost of the diagnosis associated with each patient with CHC with the alert system would decrease to €1,109 compared to €19 with non-alert.

**Fig 3:**
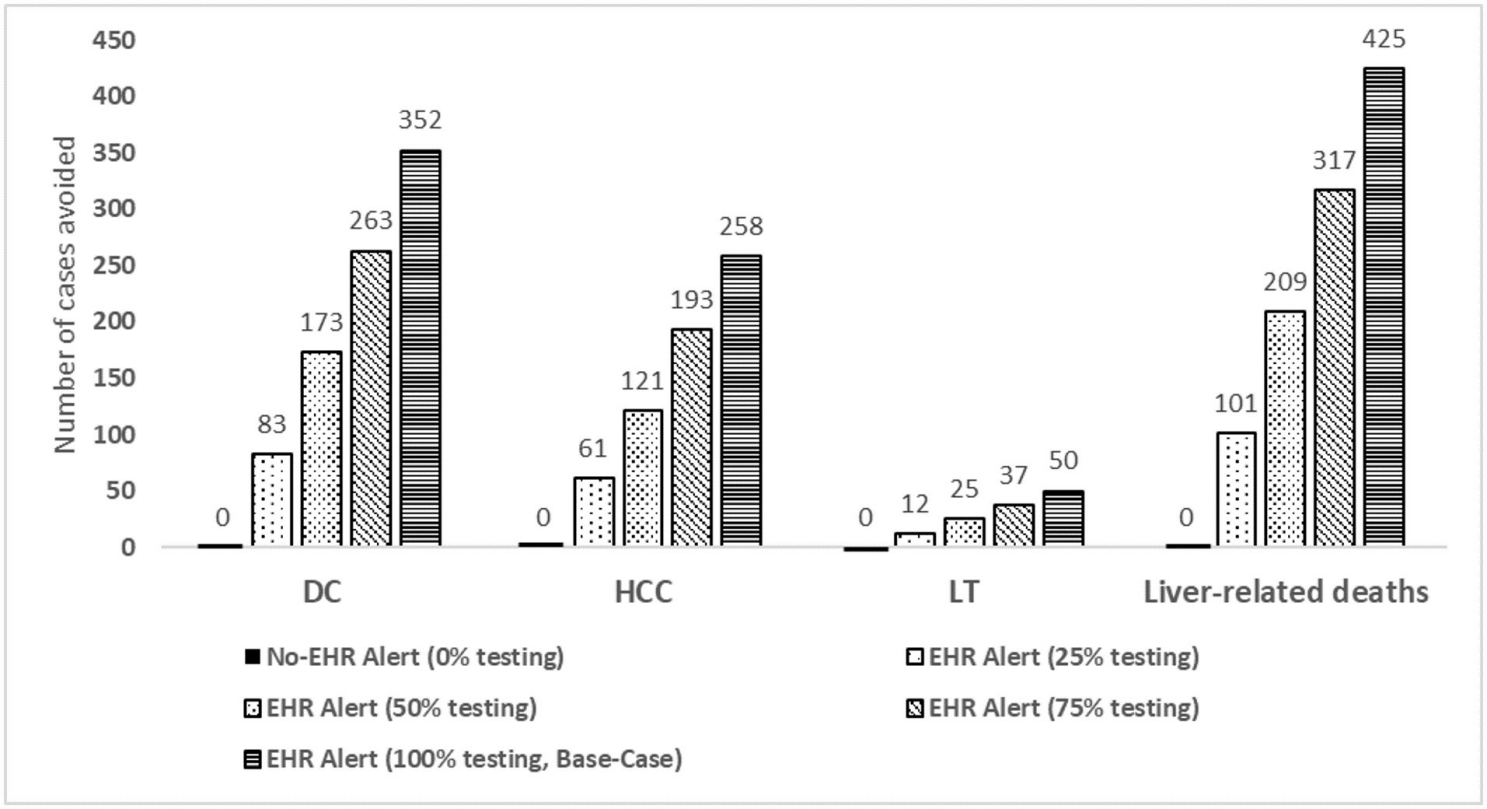
Alternative scenario 2 results: Number of cases of liver complications avoided with different percentages of screening (0–100%). DC: Decompensated Cirrhosis; F: Fibrosis stage; HCC: Hepatocellular carcinoma; LT: Liver transplant.

The results of alternative scenario 2 showed that a variation in the percentage of screened patients had an impact on the health outcomes measured in the number of cases of liver complications ranging from 83–352 cases of decompensated cirrhosis avoided, 61–258 cases of hepatocarcinoma, 12–50 cases of liver transplantation and 101–425 of deaths due to liver causes (Fig 3).

**Fig 4:**
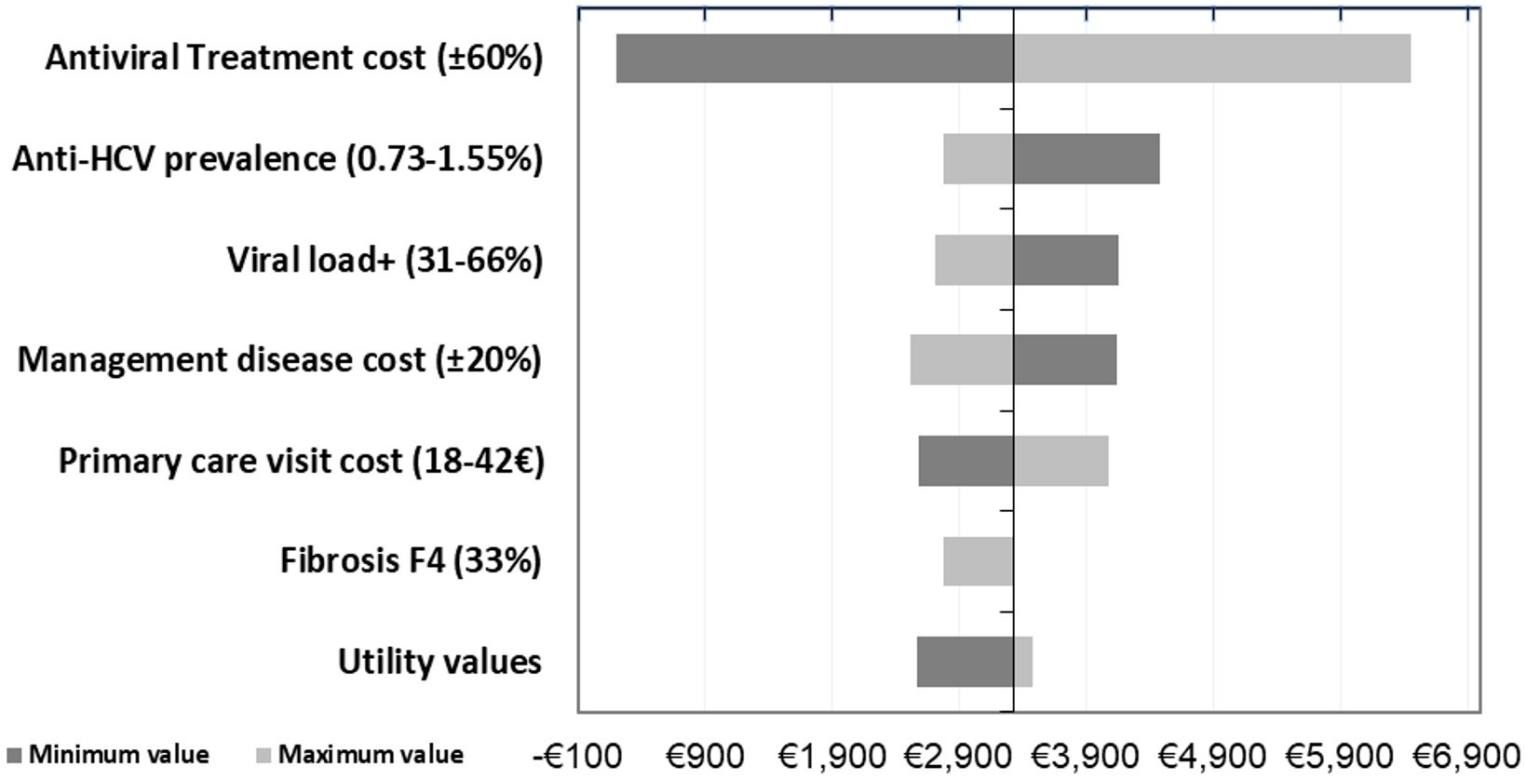
Sensitivity analysis results of the main scenario: Tornado diagram.

The results of the SA showed variations in the ICUR results of between €193 and €6,449, being the treatment cost and CHC prevalence the parameters with the greatest impact (Fig 4). In addition, a variation in the number of patients who underwent HCV serology in the non-alert arm would decrease the difference in the number of chronic patients detected, with minimal impact on the ICUR. All DSA results were well below the threshold commonly used in Spain.

## Discussion

In Spain, different measures have been adopted for the early detection of asymptomatic patients with hepatitis C and its treatment, but it is estimated that a high percentage of this people are undiagnosed and do not know their status. One way to facilitate the diagnosis of the hidden infection is to take focused actions in groups of subjects who are easily accessible and available for follow-up, as is the case of patients who attend primary care. The implementation of EHR alert systems following guidelines established by previously established health authorities [3] for the detection of asymptomatic patients in this area would imply an increase in the detection of hidden cases and their subsequent access to highly effective treatments. This type of measure has already been evaluated in other countries, with clinical results that contribute to achieving the goal of eliminating the disease [23–26].

In Spain, different strategies have been implemented by several autonomous communities, in order to increase the detection of patients with hepatitis C [5,22,27]. In the Valencian Community, one study demonstrated an increase in the detection and access to treatment of viremic patients who were unaware of their status of HCV infection in relation to a previous campaign of information and subsequent sending of letters to the general population offering serological testing for HCV [5].

From a clinical and economic point of view, with the objective of ensuring the sustainability of health systems, considering the results obtained in the last survey of HCV seroprevalence conducted in Spain [4], screening strategies conducted in general population should implemented in the highest-prevalence group (usually greater than 40 yr. old), without forgetting high-risk groups. Considering this premise, the present study focused on performing HCV serology only for the population born between 1955 and 1975 which is the group with the highest prevalence in the Valencian Community. However, consistent with the goals of eliminating HCV infection established by the WHO [1], it would also be necessary to establish complementary strategies to implement age-based EHR alert, directed at risk groups that do not routinely attend primary care consults, such as men who have sex with other men and injected drug users [28–30]. In addition, the simplification of treatments and the decrease in their price in recent years have made strategies to detect infection cost-effective [31].

The population with hepatitis C has a high risk of progression to advanced stages of liver disease, with the consequent development of complications and extrahepatic manifestations that lead to the loss of health and quality of life. On the other hand, hidden HCV infection is especially harmful to the population due to the possibility of transmitting the virus to other people due to lack of awareness. In our study, the loss of health showed the high burden of disease associated with mortality and liver complications that patients would develop as a result of non-detection if the EHR alert was not implemented, while the loss of quality of life was reflected in the increase in QALY gained with the implementation of the alert. Although the analysis did not evaluate the transmission of the infection, there are studies that show that detecting and treating patients with CHC decreases the risk of transmission [30]. Therefore, further studies should be performed to obtain information on health outcomes and the economic impact of preventing virus transmission in the study population.

When the economic results associated with the diagnosis are analysed, the total costs of implementing an EHR alert are higher than the costs of not using an alert; nonetheless, the EHR alert is an efficient strategy. This increase in costs is due to difference in the population with possible infection detected through the alert and the need to proceed with research to determine whether the disease is present and the administration of treatment if it is confirmed. On the other hand, it must be appreciated that the cost of the first visit to primary care was included in the main analysis, being conservative. However, this cost could be ignored since the serology performed derived from the operation of the alert system is requested during a primary care visit for different causes than hepatitis C. Not including this cost has a great impact on the results of the analysis, as reflected in the alternative scenario, increasing the efficiency of the EHR alert system by age.

An important premise of the analysis was the assumption that one hundred percent of the patients are screened with the alert system. This premise was evaluated in an alternative scenario with different screening ranges, considering that the performance of the serology depends on the involvement of the physicians and the patients, and in routine clinical practice, medical forgetfulness or patient refusal may occur.

Not taking actions that increase detection would not generate short-term costs to the health system but would generate increased costs in the medium and long term, when the undetected disease and therefore not treated, progresses and patients develop hepatic [8,9] and extrahepatic complications [32,33]. In addition, we should consider the social costs associated with CHC, which were not considered in this study [10].

### Conclusions

Measures aimed at identifying patients with undiagnosed HCV infection are necessary, especially within the framework of the WHO’s proposal to eliminate HCV. In this sense, the present analysis shows that the implementation of an age-based EHR alert in primary care for the detection of patients with CHC would be an efficient measure that would favour attaining the goal of eliminating the disease and would significantly reduce the incidence of hepatic and extrahepatic complications and mortality.
